# Acute biliary events during anti-tuberculosis treatment: hospital case series and a nationwide cohort study

**DOI:** 10.1186/s12879-018-2966-3

**Published:** 2018-02-01

**Authors:** Lih-Yu Chang, Chih-Hsin Lee, Chia-Hao Chang, Ming-Chia Lee, Meng-Rui Lee, Jann-Yuan Wang, Li-Na Lee

**Affiliations:** 1 Department of Internal Medicine, National Taiwan University Hospital, Hsinchu Branch, Hsinchu, Taiwan; 2 Division of Pulmonary Medicine, Wanfang Hospital, Taipei Medical University, Taipei, Taiwan; 3 School of Medicine, College of Medicine, Taipei Medical University, Taipei, Taiwan; 4 Department of Pharmacy, New Taipei City Hospital, New Taipei City, Taiwan; 5 School of Pharmacy, College of Pharmacy, Taipei Medical University, Taipei, Taiwan; 6 Department of Internal Medicine, National Taiwan University Hospital, #7, Chung-Shan South Road, Zhongzheng District, Taipei, 10002 Taiwan; 7 Department of Laboratory Medicine, National Taiwan University Hospital, Taipei, Taiwan

**Keywords:** Tuberculosis, Biliary event, Cholelithiasis, Cholecystitis, Cholangitis, National Health Insurance Research Database

## Abstract

**Background:**

Tuberculosis (TB) remains one of the major infectious diseases worldwide. Adverse reactions are common during TB treatment. Few reports, however, are available on treatment-related acute biliary events (ABEs), such as cholelithiasis, biliary obstruction, acute cholecystitis, and cholangitis.

**Methods:**

We first report four pulmonary TB patients who developed ABEs during anti-TB treatment. Abdominal sonography revealed multiple gall stones with dilated intrahepatic ducts in three patients and cholecystitis in one patient. To investigate the incidence of and risk factors for ABEs during anti-TB treatment, we subsequently conducted a nationwide cohort study using the National Health Insurance Research Database of Taiwan.

**Results:**

A total of 159,566 pulmonary TB patients were identified from the database between 1996 and 2010, and among them, 195 (0.12%) developed ABEs within 180 days after beginning anti-TB treatment. Logistic regression analysis revealed that the risk factors associated with ABEs are older age (relative risk [RR]: 1.32 [1.21–1.44] per 10-year increment) and diabetes mellitus (RR: 1.59 [1.19–2.13]).

**Conclusions:**

Although infrequently encountered, ABEs should be considered among patients with TB who experience abdominal discomfort with hyperbilirubinemia, especially patients who have older age or diabetes.

## Background

Tuberculosis (TB) remains one of the most deadly infectious diseases worldwide [1]. In 2015, approximately 10.4 million new cases of TB were diagnosed globally. Despite a decrease in the mortality rate in 2015 from 2000, TB caused approximately 1.8 million deaths in 2015 [1]. Although effective anti-TB drugs are readily available, the treatment of TB is not always successful because treatment-emergent adverse events—such as hepatotoxicity, peripheral neuropathy, gastrointestinal upset, hyperuricemia, optic neuritis, and cutaneous reactions [2]—often lead to treatment interruption and further dissemination of the TB bacilli.

Hepatotoxicity is the most common adverse event associated with TB treatment, with an incidence rate between 10.2% and 18.9% in Taiwan, and it is potentially life-threatening [3–6]. The risk of drug-induced liver injury during anti-TB treatment ranges from 5% to 33% according to the American Thoracic Society [7]. Clinical presentations may include low-grade fever, general malaise, poor appetite, nausea, vomiting, abdominal distension, icteric conjunctiva, and elevated serum aminotransferases and bilirubin levels. These symptoms and signs are typically indistinguishable from those of acute biliary events (ABEs) such as cholelithiasis, biliary obstruction, acute cholecystitis, and cholangitis. Delayed or incorrect diagnosis of ABEs may cause unnecessary anti-TB treatment interruption, or if left untreated, may lead to intra-abdominal complications, such as acute pancreatitis, sepsis, or perforation [8]. However, the association between ABEs and anti-TB treatment has not been elucidated thus far. Therefore, in this paper, we first report four cases of ABEs during anti-TB treatment, and subsequently, we investigate the incidence rate and risk factors of ABEs during anti-TB treatment in a nationwide TB cohort.

## Methods

This study consisted of two parts. In the first part (case series), we obtained the clinical presentations and treatment histories of patients with pulmonary TB who developed ABEs during anti-TB treatment at the National Taiwan University Hospital and its Hsinchu Branch between 2010 and 2015. In the second part (nationwide cohort), we conducted a nationwide cohort study using claims data from the National Health Insurance Research Database (NHIRD) of Taiwan to evaluate the incidence rate of ABEs during anti-TB treatment and identify risk factors for ABEs.

### Ethics statement

The Institutional Review Board of the National Taiwan University Hospital and its Hsinchu Branch approved the study (NTUH REC: 201,309,064 W; NTUH-HC REC: 105–023-E). Due to the study’s retrospective design, inform consent was deemed unnecessary.

### Identification of ABE cases in the hospital-based cohort (case series)

#### Definition of ABEs

An adverse event was considered an ABE if it met both of the following criteria: (1) abnormal liver function during anti-TB treatment; and (2) diagnosis with image confirmation, such as abdominal computed tomography or sonography.

#### Case selection

Between 2010 and 2015, a total of 3686 patients with pulmonary TB were treated at the National Taiwan University Hospital (*n* = 2704) and its Hsinchu Branch (*n* = 982). Among them, 2879 (78.1%) had culture-confirmed pulmonary TB. Liver function (aspartate aminotransferase [AST], alanine aminotransferase [ALT], and total bilirubin) was evaluated at baseline and at least once during anti-TB treatment in 3675 (99.7%) patients, and significant elevation in AST and ALT levels was noted in 416 (11.3% of 3675) patients. The medical records of these 416 patients were reviewed to determine whether ABE was responsible for abnormal liver function in these patients. The diagnosis of ABE was also confirmed using imaging modalities.

### Analysis of nationwide cohort

#### Definition of ABEs

An adverse event was considered an ABE if it met both of the following criteria: (1) diagnosis of cholelithiasis (ICD-9-CM code 574), acute cholecystitis (ICD-9-CM code 575.0–575.2), cholangitis (ICD-9-CM code 576.1), or obstruction of biliary tract (ICD-9-CM code 576.2); and (2) the patient had received at least one of the examinations and management therapies listed in Table 1. The primary outcome was ABEs that occurred within 180 days after commencement of anti-TB treatment.

**Table 1:** Examinations and management of acute biliary events

	Examination or Management	NHIRD code
Non-invasive Study	Cholescintigraphy	26040B
	Oral cholecystography	33020B
	Intravenous choledochocystography	33021B
Invasive Study	Transduodenal choledochoscopy	28032B
	Percutaneous transhepatic choledochoscopy	28036B
	Endoscopic retrograde cholangiopancreatography	33024B
	Percutaneous transhepatic cholangiography	33025B
Drainage	Fiber choledochoscopy, percutaneous via T-tube or other tract	28034B
	T-tube cholecystography	33022B
	Percutaneous transhepatic cholangiography-drainage	33026B
	Percutaneous gall bladder drainage	33106B
	Endoscopic retrograde biliary drainage	56020B
	Endoscopic nasobiliary drainage	56021B
	Choledochoscopy and choledochotomy	56034B
	Choledochotomy with T-tube drainage	75208B
	Choledocholithotomy with T-tube drainage	75209B
Stone Removal	Fiber choledochoscopy, percutaneous via T-tube or other tract, with removal of stones	28008B
	Fiber choledochoscopy, intraoperative, with removal of stones	28035B
	PTCD-stone removal	33083B
	Choledocholithotomy (transduodenal)	75202B
Operation		
Traditional	Fiber choledochoscopy, intraoperative	28007B
	Operative cholangiography	33023B
	Cholecystostomy	75201B
	Cholecystectomy	75203B
	Choledochojejunostomy	75204B
	Cholecystoenterostomy	75205B
Laparoscopic	Laparoscopic cholecystectomy	75215B
	Laparoscopic choledocholithotripsy	75218B

#### Case selection

**Fig. 1:**
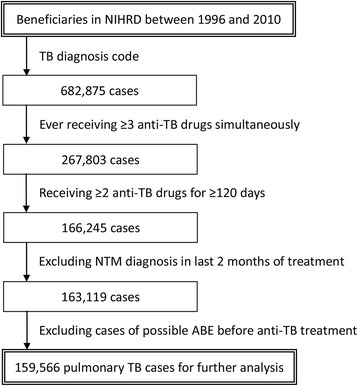
Flowchart of selection of pulmonary TB cases in the nationwide cohort

We identified patients in the NHIRD who had been diagnosed with pulmonary TB from 1996 to 2010 (Fig. 1). Active TB was defined by the following criteria: (1) at least two outpatient records or one inpatient record with a compatible diagnosis; (2) one prescription of at least three anti-TB medications; and (3) receiving two or more anti-TB medications simultaneously over a period of 120 days within a 180-day period [9]. Patients who were diagnosed with nontuberculous mycobacterial infection (ICD-9-CM code 031) during the last 60 days of anti-TB treatment were excluded. Patients who met any of the ABE diagnostic criteria before anti-TB treatment were also excluded. Date of treatment completion was defined as the last date on which two or more anti-TB drugs were taken simultaneously without receiving any anti-TB medication in the subsequent 60 days.

#### Comorbidity and income status

Comorbid conditions—comprising diabetes mellitus (DM), chronic obstructive pulmonary disease (COPD), malignancy, end-stage renal disease, connective tissue disease, acquired immunodeficiency syndrome, liver cirrhosis, transplantation, pneumoconiosis, and low-income status—at the time TB diagnosis were identified by using previously published definitions [9, 10].

#### Statistical analyses

Intergroup differences were assessed by using independent-sample *t* tests for continuous variables and chi-square or Fisher exact tests for categorical variables. The risk factors for ABEs in patients with pulmonary TB were evaluated using logistic regression analyses. Only variables with a two-sided *p* < 0.05 were included in the final model. All analyses were performed using IBM SPSS Statistics Version 22 (IBM Corp., Armonk, NY, USA).

## Results

### Case series in the hospital cohort

#### Case 1

A male patient in his 80s with comorbid, medically controlled hypertension and DM received standard four-drug anti-TB treatment (isoniazid, rifampin, ethambutol, and pyrazinamide) for his smear-positive, culture-confirmed pulmonary TB. He experienced fever and chills after one week of treatment. Laboratory examinations showed elevated AST (212 IU/L) and ALT (375 IU/L) and direct-type hyperbilirubinemia (total bilirubin: 2.81 mg/dL; direct bilirubin: 2.57 mg/dL). Abdominal sonography revealed the presence of multiple gall stones and dilated intrahepatic ducts. Anti-TB treatment was discontinued and ceftazidime was prescribed. One month later, the patient underwent a laparoscopic cholecystectomy procedure, followed by the resumption of anti-TB treatment. Thereafter, ABEs were not noted.

#### Case 2

A male patient in his 50s with comorbid, medically controlled DM and COPD was diagnosed with pulmonary TB based on a histology report of a transbronchial lung biopsy and a mycobacterial culture of bronchial washing sample. The first 3 weeks of four-drug anti-TB treatment were uneventful, but after 4 weeks of treatment, he experienced nausea and vomiting. Laboratory tests revealed elevated transaminase (AST: 144 IU/L and ALT: 358 IU/L). A minimal amount of ascites in the right subdiaphragmatic area was observed using abdominal sonography. A small amount of ascites was also observed in the perigallbladder, right anterior pararenal, and pelvic space one week later using abdominal computed tomography. The patient was diagnosed with acute cholecystitis. Anti-TB treatment was interrupted and cefmetazole was administered. After his adverse reactions had resolved, anti-TB treatment was successfully resumed.

#### Case 3

A man in his 50s with comorbid hypertension and COPD, which were under irregular medical control, was diagnosed with smear-positive, culture-confirmed pulmonary TB. Two weeks prior to the commencement of standard anti-TB treatment, he had pneumonia with respiratory failure and septic shock. Laboratory tests demonstrated elevated transaminase levels, but normal total and direct bilirubin (AST: 144 IU/L; ALT: 358 IU/L; and total bilirubin: 0.52 mg/dL). Neither gallbladder stones nor bile duct stones were identified using computed tomography (slide thickness: 5 mm; interval: 1.2 mm). The patient was treated with courses of antibiotics, which improved his respiratory condition, hemodynamic condition, and aminotransferase levels (AST: 51 IU/L; ALT: 23 IU/L). The standard four-drug anti-TB treatment was started subsequently. However, after receiving anti-TB treatment for two weeks, his bilirubin level was increased, with a mild elevation in transaminase level (AST: 77 IU/L; ALT: 49 IU/L; total bilirubin: 4.57 mg/dL; and direct bilirubin: 3.84 mg/dL). Anti-TB treatment was halted. Multiple cholelithiasis with a dilated intrahepatic duct was diagnosed using abdominal sonography. Soon after the patient underwent the sonography procedure, he experienced another episode of pneumonia with septic shock and respiratory failure. Although bilirubin and transaminase both improved after interruption of anti-TB treatment for 10 days (AST: 61 IU/L; ALT: 38 IU/L; total bilirubin: 1.72 mg/dL; and direct bilirubin: 1.37 mg/dL), the general condition of the patient deteriorated gradually. The patient died of refractory septic shock.

#### Case 4

A previously healthy male patient in his 20s was diagnosed with smear-positive, culture-confirmed pulmonary TB and TB pleurisy. No cholelithiasis was noted in abdominal sonographs obtained 2 years prior to the diagnosis of TB. The standard four-drug anti-TB treatment improved his TB clinical symptoms, including fever, chest pain, and left pleural effusion; however, abnormal liver function was noted (AST: 303 IU/L; ALT: 499 IU/L; and total bilirubin: 0.56 mg/dL) after 6 weeks of treatment, which resolved (ASL: 26 IU/L; ALT: 34 IU/L; total bilirubin: 0.1 mg/dL) after the interruption of anti-TB treatment. In the 18th week, anti-TB treatment with isoniazid, rifampin, and ethambutol was resumed. However, the patient experienced acute abdominal cramping pain in the following week. His aminotransferase and bilirubin level did not increase (AST: 22 IU/L; ALT: 17 IU/L; and total bilirubin: 0.57 mg/dL). Multiple small cholelithiases were discovered using abdominal sonography. Rechallenge with anti-TB treatment was complicated by recurrent abdominal cramping pain immediately after taking the anti-TB drugs. Because gastroenterologists suggested a possible diagnosis of intermittent biliary obstruction due to a passing stone, he received conservative management. The anti-TB treatment was successfully completed in the ninth month after TB diagnosis.

The demographic characteristics and clinical course of these four patients are summarized in Table 2.

**Table 2:** Characteristics and clinical course of the cases of acute biliary events

	Case 1	Case 2	Case 3	Case 4
Age (decade of life)	9th	6th	6th	3rd
Sex	male	male	male	male
Co-morbidity	DM	DM, COPD	HTN, COPD	Nil
Anti-TB regimen before ABE	HREZ	HREZ	HREZ	HRE
Presenting symptoms	Fever/Chills	Nausea/Vomiting	Fever	Abdominal pain
Peak ALT level (U/L)^a^	375	303	16	499
Peak AST level (U/L)^b^	212	223	98	303
Peak total bilirubin level (mg/dL)^c^	2.81	1.53	1.69	0.57
Diagnosis of ABE	Cholecystitis	Cholecystitis	Cholelithiasis with biliary obstruction	Cholelithiasis with biliary obstruction
Onset of ABE	1 week later	4 weeks later	2 weeks later	18 weeks later
Treatment of ABE	LC	Antibiotics	Antibiotics	Antibiotics

*ABE* Acute biliary event, *ALT* Alanine aminotransferase, *AST* Aspartate aminotransferase, *COPD* Chronic obstructive pulmonary disease, *DM* Diabetes mellitus, *E* Ethambutol, *H* Isoniazid, *HTN* Hypertension, *LC* laparoscopic cholecystectomy, *R* Rifampin, *TB* Tuberculosis, *Z* Pyrazinamide

^a^normal range of ALT: 2–32 U/L

^b^normal range of AST: 10–30 U/L

^c^normal range of total bilirubin: 0.2–1.2 mg/dL

### NHIRD cohort

From the beneficiaries listed in the NHIRD between 1996 and 2010, a total of 682,875 cases with diagnosis of pulmonary TB were identified (Fig. 1). A total of 159,566 cases that met the diagnostic criteria for pulmonary TB were included for further analysis. Subsequent analysis identified 195 (0.12%) patients who developed ABEs within the initial 180 days of treatment. The patients who did not develop ABEs comprised the non-ABE group. The clinical characteristics of the ABE and non-ABE groups—their age, sex, and underlying comorbidities—are summarized in Table 3. The mean age of the patients in the ABE and non-ABE groups was 66.2 ± 13.5 years and 56.6 ± 19.7 years, respectively (*p* < 0.001). Men accounted for 67.7% of the ABE group and 68.6% of the non-ABE group (*p* = 0.075). Of the underlying comorbidities and income condition, a significant difference was only obtained between the ABE and non-ABE groups regarding DM prevalence (*p* < 0.001). The duration and type of anti-TB treatment were similar between the ABE and non-ABE groups, except for rifamycin prescription during the initial 60 and 180 days after TB diagnosis and isoniazid prescription during the initial 180 days.

**Table 3:** Characteristics of patients with and without acute biliary events

	ABE group (*n* = 195)	Non-ABE group (*n* = 159,371)
Age (years)^a*^	66.2 ± 13.5	56.6 ± 19.7
Male^b^	132 (67.7)	109,399 (68.6)
Tuberculosis diagnostic year^b^		
1996–2000	60 (30.8)	48,844 (30.6)
2001–2005	73 (37.4)	59,424 (37.3)
2006–2010	62 (31.8)	51,103 (32.1)
Co-morbidity (%)		
Diabetes mellitus^b*^	63 (37.4)	38,075 (23.9)
Chronic obstructive pulmonary disease^b^	10 (5.1)	7918 (5.0)
Malignancy^b^	12 (6.2)	6712 (4.2)
End-stage renal disease^c^	2 (1.0)	1998 (1.3)
Connective tissue disease^c^	0 (0.0)	1151 (0.7)
Acquired immunodeficiency syndrome^c^	1 (0.5)	562 (0.4)
Liver cirrhosis^c^	1 (0.5)	323 (0.2)
Transplantation^c*^	1 (0.5)	137 (0.1)
Pneumoconiosis^c^	0 (0)	85 (0.1)
Low income^b^	5 (2.6)	4830 (3.0)
Total duration of anti-TB treatment	266.9 ± 99.7	260.4 ± 105.8
Intensive phase (initial 60 days)^a^		
No. of days covered by isoniazid^a^	47.7 ± 18.6	50.0 ± 11.2
No. of days covered by rifamycin^a*^	48.9 ± 13.4	51.7 ± 11.2
No. of days covered by ethambutol^a^	51.2 ± 11.4	50.8 ± 13.1
No. of days covered by pyrazinamide^a^	39.9 ± 21.3	42.5 ± 20.4
Initial 180 days		
No. of days covered by isoniazid^a*^	134.4 ± 52.2	142.4 ± 53.4
No. of days covered by rifamycin^a*^	140.1 ± 37.9	152.5 ± 32.8
No. of days covered by ethambutol^a^	145.2 ± 35.6	141.9 ± 42.2
No. of days covered by pyrazinamide^a^	67.8 ± 44.9	69.3 ± 45.8

Data are presented as number (%) or mean ± standard deviation

^a^Compared using an independent-sample *t* test

^b^Compared using a chi-square test

^c^Compared using a Fisher exact test

*p < 0.05 for the comparison between the ABE and non-ABE groups

**Fig. 2:**
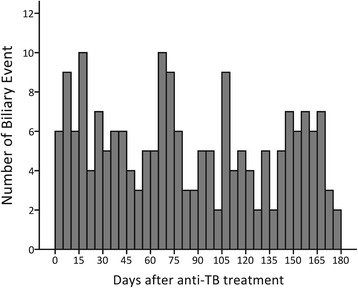
Time of onset of acute biliary events

The onset of ABEs was evenly distributed within the 180-day period (Fig. 2). The median number of days of onset was 76 days (IQR: 76–133) after initiation of anti-TB treatment. Seventy-two (36.9%) events occurred within the first 60 days of anti-TB treatment, 65 (33.3%) within the next 60 days, and the remaining 58 (29.7%) within the final 60 days. Among the 195 patients, 132 (67.7%) received surgical intervention, which was either a traditional operation (*n* = 72) or laparoscopic surgery (*n* = 60) (Table 4). Twenty-four (12.3%) received nonsurgical stone removal, and 66 (33.3%) drainage.

**Table 4:** Management and intervention for acute biliary events in the nationwide cohort

	ABE Group (n = 195)
Non-invasive study (excluding abdominal sonography)	5 (2.6%)
Invasive study	63 (33.3%)
Drainage	66 (33.8%)
Stone removal	24 (12.3%)
Operation	132 (67.7%)
Traditional operation	72 (36.9%)
Laparoscopic operation	60 (30.8%)

Risk factors associated with ABEs were assessed using multivariable logistic regression analysis. We discovered that age (*p* < 0.001; relative risk [RR]: 1.32 [1.21–1.44] per 10-year increment) and DM (*p* < 0.001; RR: 1.59 [1.19–2.13]) were significantly associated with occurrence of ABEs.

## Discussion

To our knowledge, this was the first study to investigate ABEs during anti-TB treatment. The incidence rate of ABEs during anti-TB treatment in the case study cohort was 0.11% (4/3686), which was similar to the finding in the nationwide cohort (0.12%). ABEs do not appear to onset at a particular time. The risk factors for ABEs are older age and comorbid DM, with the latter occurring in half of the patients in the case series.

Because ethambutol and streptomycin are the only first-line TB therapies that are not associated with hepatotoxicity, abnormal liver function remains common during anti-TB treatment. Transient asymptomatic elevation of aminotransferase levels was observed in 10% to 20% of patients who have received isoniazid [11]. Furthermore, transient asymptomatic hyperbilirubinemia was detected in 0.6% of patients receiving rifampin [2]. The incidence of symptomatic and severe hepatotoxicity varies between different studies, ranging from 5% to 33% [7]. However, the clinical presentations of hepatotoxicity are typically indistinguishable from those of ABEs. Distinguishing hepatotoxicity from ABEs has not been investigated in this field. Therefore, when a patient who is treated for TB presents symptoms of an ABE—right upper quadrant pain, nausea, and vomiting with hyperbilirubinemia— the patient may be misdiagnosed with drug-induced hepatitis by the primary care physician. If the ABE is mild or self-limited, such as a passing stone, the physician may attribute the patient’s recovery to discontinuation of anti-TB treatment, further delaying the diagnosis of an ABE. Because some ABE cases should be managed aggressively with invasive procedures (more than two-thirds in the present nationwide cohort), delayed diagnosis may increase morbidity and mortality [12]. The findings of this study emphasize that although ABEs are not common, increasing awareness of these potential complications and practicing differential diagnosis are crucial for early diagnosis and proper management of ABEs.

From the nationwide cohort in this study, we discovered that older age and comorbid DM are two significant risk factors for ABEs during anti-TB treatment. These findings are similar to those of a previous multicenter study performed in Italy [13], which discovered that older age and DM are both independent risk factors for cholelithiasis. Epidemiological studies on the risk of gallbladder disease among patients with DM have yielded inconsistent results. In a case–control study [14], the prevalence of DM was significantly higher in patients with gallstone than in the control group (11.6% vs. 4.8%, odds ratio 2.55 [1.39–4.67]). In studies demonstrating a positive association between DM and gallbladder disease or gallstones, the risk ratios have been reported to lie in the range 1.68–2.09 [15–18]. Other studies, however, have not found an association [19, 20]. The results of a recent meta-analysis suggested that a diagnosis of DM may increase the relative risk of gallbladder disease by 56% [21].

Patients with ABEs during anti-TB treatment may have asymptomatic cholelithiasis before treatment. When treatment begins, isoniazid and rifampin may inhibit the bile salt export pump [22] and accelerate the formation of cholesterol stone [23], with the previous asymptomatic stone used as a nucleus, resulting in a higher risk of ABEs. Cholelithiasis may be caused by either cholesterol gallstones, which are a mixture of cholesterol, bile salt, and phospholipids, or pigmented gallstones. The relative proportion of each component predicts if the mixture of cholesterol, bile salt, and phospholipids forms crystals, micelles, or vesicle micelles [24]. Crystal formation is associated with higher proportion of bile salt. The crystal becomes the nucleus of a cholesterol gallstone. Insulin treatment has been shown to increase the biliary saturation index, resulting in cholesterol precipitation and gallstone formation [25, 26].

Another possible mechanism for the increased risk of an ABE in patients with DM may be decreased gallbladder motility as a result of denervation [27, 28]. The underlying pathogenesis is still unclear. One possibility is fewer cholecystokinin receptors on the gallbladder wall due to diabetic autonomic neuropathy leading to poor response to cholecystokinin stimulation [27]. In addition to impaired cholinergic innervation, cholecystoparesis may result from increased dopaminergic activity [29–32].

We discovered that duration of rifamycin use in the ABE group was significantly shorter than that in the non-ABE group. This finding is most likely to be confounded by indication. Among the first-line anti-TB drugs, only rifampin has been reported to be associated with cholestasis, which typically occurs in the first month of treatment [7]. Rifampin inhibits the major bile salt exporter pump and blocks bilirubin uptake [33]. In addition, rifampin competitively affects the clearance of bilirubin from the sinusoidal membrane or canaliculular level in a dose-dependent manner [34, 35]. Therefore, when the bilirubin level increases, primary care physicians tend to discontinue rifamycin treatment first. However, it was impossible to confirm the effect of rifampin on the development of ABEs in this retrospective study. Because it is unethical to treat TB without using rifamycin, a prospective interventional study comparing different doses of rifampin could be used to address this issue.

The present study had some limitations. First, because of the retrospective design of the study, the incidence of ABEs was probably underestimated in the hospital-based cohort because of missing mild and transient events. However, the effect of this underestimation may not be large because serum transaminase and bilirubin assessments in the second, fourth, and eighth weeks of treatment are recommended by TB treatment guidelines in Taiwan [36], and the percentage of follow-up tests performed for liver function in the hospital-based cohort was > 90%. Second, because the NHIRD cohort study was based solely on claims data, ABE incidence may have been underestimated because of noncomprehensive data. Finally, the causal relationships between ABEs, rifampin treatment, and comorbid DM were not addressed. Further prospective studies are necessary to clarify these issues.

## Conclusion

Although uncommon, ABEs develop in approximately 0.12% of patients with pulmonary TB receiving anti-TB treatment. Clinicians should be aware of this potential complication of anti-TB treatment and maintain high clinical suspicion for patients with pulmonary TB who experience abdominal discomfort during anti-TB treatment, especially elderly patients and those with DM.

## Abbreviations

ABE: Acute biliary event
ALT: Alanine aminotransferase
AST: Aspartate aminotransferase
COPD: Chronic obstructive pulmonary disease
DM: Diabetes mellitus
NHIRD: National Health Insurance Research Database
RR: Relative risk
TB: Tuberculosis
